# Fabrication of graphene-based quantum Hall networks and influences of partial star-mesh recursion

**DOI:** 10.1088/2053-1591/ae7250

**Published:** 2026-06-01

**Authors:** D S Scaletta, N T M Tran, M Musso, V Ortiz Jimenez, H M Hill, D G Jarrett, M Ortolano, C A Richter, D B Newell, A F Rigosi

**Affiliations:** 1 Department of Physics, Mount San Jacinto College, Menifee, CA 92584, United States of America; 2 Physical Measurement Laboratory, National Institute of Standards and Technology (NIST), Gaithersburg, MD 20899, United States of America; 3 Joint Quantum Institute, University of Maryland, College Park, MD 20742, United States of America; 4 Department of Electronics and Telecommunications, Politecnico di Torino, Torino 10129, Italy

**Keywords:** graphene, quantum Hall effect, fractal, resistance metrology

## Abstract

This work presents a substantial advancement on how one may develop high-resistance quantized Hall array resistance standards (QHARS) by using star-mesh transformations for element count minimization. More specifically, this work introduces a generalized mathematical reconciliation to recover exact *effective* quantized resistances found by simulation and measurement. Furthermore, this work explores the concept of fractal dimension, clarifying the benefits of both full and partial recursions in QHARS devices. Three different partial recursion cases are visited for a near-1 GΩ QHARS device. These partial recursions, analyzed in the context of their fractal dimensions, offer increased flexibility in accessing desired resistances within a specific neighborhood of values compared to full recursion methods, though at the cost of the number of required elements.

In the field of electrical metrology, graphene may be grown epitaxially and has been recently developed into prototype devices that can provide incredibly accurate quantized resistance due to its robust quantum Hall effect [1], and most epitaxial-graphene-based (EG-based) devices that serve as a standard for electrical resistance operate at the ν=2 Landau level ($R_{\text{H}}=\frac{1}{2}\frac{h}{e^{2}}\approx 12906.4037\Omega$). Since only one value of quantized electrical resistance is typically accessed from these types of devices, it may be challenging for primary standards laboratories or National Metrology Institutes to calibrate resistances that are more than a couple of orders of magnitude different than the measured quantum Hall resistance. Since the start of the efforts in using EG-based devices, there have been two main approaches in the literature for removing these single-value limits. The first approach uses quantum Hall array resistance standards (QHARS), and these work by connecting multiple Hall elements in series or parallel [2, 3]. The second approach can use p-n junctions, which typically yield rational multiples $R_{\text{H}}$ [4].

Obtaining a large total area of high-quality EG, currently limited to the centimeter scale [5], remains a crucial step for designing any QHARS device requiring many individual Hall bar elements. With the lateral dimensional constraints, there is an inherent limitation on the total number of attainable QHARS elements through fabrication. As an example, an array that has 500 elements in series yields a maximum quantized resistance of approximately 6.5MΩ, and this order of magnitude is much smaller than the range of resistances currently calibrated [6]. Future QHARS devices may use star-mesh transformations that can achieve resistances at the highest levels of necessity, such as at the MΩ (shown experimentally in [7]) and GΩ levels (hypothetically feasible per the recent foundational work in [8] and sister study in [9]).

This work continues to expand on a recently established, mathematical framework seeking to minimize the required number of elements in a QHARS to achieve high effective quantized resistances [8]. One of the key points of the framework was to optimize a star-mesh QHARS device design using full and symmetric recursion principles, and though this approach allows one to rapidly calculate an optimal device design, the designed device itself may hold a slightly different value than desired because of approximations inherent to the initial framework. Those discrepancies are understood and explained herein. Presented data from an approximately 1GΩ QHARS device also support the underlying principles of this work.

In addition to the framework adjustments to full recursions, which are one form of pseudofractal in the context of the topology of the QHARS device design, other example pseudofractals are explored to reveal the benefits of partial recursions in QHARS device designs. All pseudofractals are examined via their Minkowski–Bouligand dimension (MBD) [9], a measure that allows one to quantify complexity and correlate it with the flexibility of a QHARS device design when subjected to minor modifications. The analysis presented here offers strong advantages to a metrologist because such information may help prevent a full QHARS device from being rendered unusable due to a single grounded Hall element failure. All analyses are applicable to material systems that exhibit the quantum Hall effect, as well as artifact standard resistors. It should be noted here that although [8] laid out foundational star-mesh analysis for EG-based QHARS devices, it left three critical questions unaddressed: (1) how to mathematically bridge the gap between approximate and exact device values without relying solely on simulations; (2) how to utilize partial recursions to access specific resistance values that full symmetric recursions completely skip; and (3) how to quantitatively assess the fault tolerance of these complex designs.

For the purposes of this study, the procedure from [5] was consulted to fabricate QHARS devices, which started out as square silicon carbide chips (with lateral side dimensions each measuring 7.6 mm). The chips were exposed to high heat to induce silicon sublimation [5]. One may summarize the full device fabrication process in terms of growth, fabrication, and post-fabrication/packaging. Finished samples of EG were checked with an optical microscope, and then each chip underwent fabrication of NbTiN electrical contacts [5]. For resistance measurements, QHARS devices were immersed in a cryostat environment at approximately 2 K, and the setup used for obtaining precision resistance was a dual source bridge (DSB) [7]. Also, for the purposes of this work, one will need to rely on previously defined mathematical definitions as set forth in equations (1) and (2) below (note that $i\neq j;q\equiv \frac{R}{R_{\text{H}}}$ and $q:q\in Z^{+};M$ is the recursion number; ξ is the number of grounded branches; $D_{\text{T}}$ is the total number of elements; the single-indexed $q_{M:i}$ is the intended number of fabricated elements [8]):

(1) $$q_{ij}=q_{i}q_{j}\sum_{\alpha =i}^{N}\frac{1}{q_{\alpha }}$$

(2) $$D_{\text{T}}\left(M,\xi ,q_{M:ij}\right)=\frac{2^{M}}{\xi }{\left(\xi q_{M:ij}^{\left(\text{approx}\right)}+1\right)}^{2^{-M}}-\frac{2^{M}}{\xi }+\left(2^{M}-1\right)\xi .$$

It will benefit the reader to recall that the indices i and j refer to the specific terminals or nodes of a star resistance network. When there are two indices for a term, that term represents the effective resistance between two distinct terminals (again i and j) in the equivalent mesh network following a star-mesh transformation. Such terms require two indices because it characterizes the calculated resistance across a specific pair of terminals (like in figure 1(b)). Whenever single or dual-index quantities have subscripts with a prefix of ‘M:’, it should be noted that these quantities have values that evolve during the course of intermediate mathematical steps, as best demonstrated by the illustration in figure 1(a). The prefix is meant to serve as a bookkeeping mnemonic for those utilizing these enhanced predictive frameworks. Furthermore, the superscript of ‘(approx)’ in equation (2) just reflects the fact that the older framework from [8] only approximates the total effective resistance and will not match a simulation of the same network.

This function of three variables was used as a starting point for device design optimization and was derived using first principles in electrical networks [10]. In [8], it was found that for a *desired* quantized resistance (the dual-indexed and *effective* number $q_{M:ij}^{\left(\text{approx}\right)}$), a global minimum in equation (2) would correspond to values of M and ξ, typically as non-integers. The next step would be to round M and ξ to the nearest integer and calculate $q_{M:i}$ in equation (3), followed by rounding of $q_{M:i}$ to the nearest integer. Now with all three integers determined, one may find the *effective* (near-exact) number of elements represented by $q_{M:ij}^{\text{(near}-\text{exact)}}$ (two indices):

(3) $$q_{M:ij}^{\left(\text{near}-\text{exact}\right)}=\frac{1}{\xi }{\left(\xi q_{M:i}+1\right)}^{2^{M}}-\frac{1}{\xi }.$$

When the *effective* (near-exact) number of elements was calculated in [8], it provided a more accurate numerical value for the expected quantized resistance output of the QHARS with the corresponding design parameters M,ξ, and $q_{M:i}$ (all integers). The issue is that this optimization process, centered on minimizing element count, contained some minor necessary approximations, without which one would not have the straightforward ability to calculate a device design within the neighborhood of a desired value of quantized resistance. The minor approximation, in short, is the exclusion of virtual resistors that contribute to the overall network when utilizing the star-mesh transformation. Examples of how these virtual resistances contribute are shown in figure 1. One may argue that the optimization accomplished its objective of providing the proper neighborhood of quantized resistance in an efficient manner and that the analog electronic circuit simulator LTspice can provide the exact resistance expected [11]; however, it would be appropriate to summarize how the exactness may be recovered mathematically.

The illustration in figure 1(a) shows that, for an example final M=3 device design, some virtual resistances $\left(q_{m:\neg ij}\right)$ contribute to the overall resistance network. Here, one defines $q_{m:\neg ij}$ as the virtual (and still technically *effective*) resistance branches that emerge after a star-mesh transformation is applied (the symbol ¬ij, or ‘not ij’, indicates any other *effective* resistance that does contribute to the dominant *effective* resistance that is desired—see the bottom of figure 1(b)). After the full device design optimization process, the quantity $q_{M:i}$ always reflects an exact number of elements, whereas every other form of q represents an *effective* resistance.

With every star-mesh transformation contributing virtual resistances, calculating $q_{m<M:ij}^{\text{(exact)}}$ becomes more cumbersome, and one way to simplify the manual procedure of recalculating $q_{0:ij}^{\text{(exact)}}$, which is the quantity one should expect to measure from the device, is to treat all ξ and $q_{m:\neg ij}$ branches as parallel branches in a single Y-Δ network (as in figure 1(b)). These conditions allow one to formulate the mathematical corrections as:

(4) $$q_{m:ij}^{\left(\text{exact}\right)}=2q_{m+1:ij}^{\left(\text{exact}\right)}+\xi {\left(q_{m+1:ij}^{\left(\text{exact}\right)}\right)}^{2}+2\xi \frac{{\left(q_{m+1:ij}^{\left(\text{exact}\right)}\right)}^{2}}{q_{m+1:\neg ij}}.$$

It is important to note that the last term of equation (4) vanishes for $q_{m=M:ij}^{\left(\text{exact}\right)}$ since it does not represent a virtual (*effective*) resistance. Furthermore, the definition of $q_{m:\neg ij}$ becomes straightforward from equation (1):

(5) $$q_{m:\neg ij}=q_{m+1:ij}^{\left(\text{exact}\right)}+2{\left\{\xi +\sum_{x=m+1}^{M-1}\left[\frac{2}{q_{x:\neg ij}}\right]\right\}}^{-1}.$$

Note that the recursive nature of equation (4) and the condition established in equation (5) allow for the recovery of exactness for any arbitrary recursion depth M. This correction is a generalized approach applicable to any QHARS topology utilizing recursive star-mesh transformations, including partial recursion cases to be explored later. With all terms now well-defined, one can apply this method to an example device design.

The desired *effective* quantized resistance was 1GΩ (i.e. ‘quantized’ meaning elements fully exhibiting the quantum Hall effect and thus providing the same exact resistance to within the measurement errors), and the QHARS design optimization yielded the device shown in figures 2(a) and (b) (with the corresponding parameters M=2,ξ=3, and $q_{M:i}=7$). Using equation (3), one finds that $q_{M:ij}^{\text{(near–exact)}}=1.007797\;\ldots \;\text{G}\Omega$. The LTspice simulation for the device matched the exact value found via equation (4): $q_{0:ij}^{\left(\text{exact}\right)}=1.095069\;\ldots \;\text{G}\Omega$. To support the simulation, precision measurements of the device were performed via DSB, allowing for sensitivities on the order of μΩ/Ω [12, 13]. See supplemental material for more context and information about calibrations and how a DSB functions [14].

In short, a DSB is a modified Wheatstone bridge allowing one to compare two resistances with incredible precision. The DSB data are shown in figures 2(c) and (d) to confirm the quantization (i.e. functionality) of the QHARS device using a 1GΩ and 10GΩ standard resistor, respectively. In the context of how a bridge functions, the QHARS device was treated as a mystery resistor, a series of measurements were performed to estimate the value of the QHARS. At each voltage, four distinct data points were collected, where each point was the result of a measurement integration time on the order of an hour. Those four values were then averaged and a standard deviation was computed. The resulting overall QHARS resistance (at each voltage) showed a relative deviation (from the exact QHARS output value) of approximately 5 parts in 10^6^ (at 6 V) and 15 parts in 10^6^ (at 10 V). So, to summarize, figure 2(c) shows the deviation from nominal $\delta R_{1}$ (centered around $q_{0:ij}^{\text{(exact)}}=1.095069\;\ldots \;\text{G}\Omega$) and the error bars represent a 1σ uncertainty.

As a second experimental verification process, a second, well-known 10GΩ standard resistor was treated as an unknown resistor and by using the QHARS device as the known quantity (whose value is the ideal value of about 1.095 069…GΩ), one obtains the results of figure 2(d). There, the highlighted blue is the 1σ uncertainty on the value of the standard resistor calibrated via conventional traceability chain, and the data points are collected in the same way as in (c), with error bars signifying a 1σ uncertainty. The deviation of the 10GΩ resistor value from as determined by the QHARS device from the conventionally calibrated value is defined as $\delta R_{2}^{\text{ref}}$.

Until now, the discussion has focused primarily on using full recursion to design a device in the neighborhood of a desired quantized resistance, with an option to manually calculate (or alternatively, simulate with LTspice) the exact, measurable value of the corresponding device design. One of the risks of using full recursion is that if any of the grounded branches fail, whether by poor quantization or contacting, the expected value of the device changes substantially. One way to both secure the benefit of rapidly approaching high *effective* quantized resistances and mitigate the aforementioned risk is to consider the implementation of other foundational pseudofractal designs (of which the full recursion, or case 0, is just one). A cursory examination of potential pseudofractal designs for 1GΩ reveals that more complex pseudofractals are correlated with a more rapid divergence from the neighborhood of desired resistance values in the event of a grounded branch failure (or deviation). To commence any correlation analysis between QHARS design pseudofractal complexity and some metric indicating the resilience or recoverable utility of a QHARS device, one must first mathematically derive some quantities for other pseudofractal designs, primarily by modifying the definition of M to account for different kinds of recursions. To that end, three additional cases were considered based on a previous work [9], each with a unique rule of iteration and thus a different definition of M. One should expect equation (2) to change based on the new definition.

Figure 3(a) shows the three new cases, with the first being a unidirectional partial recursion, the second being a bidirectional partial recursion, and the third being a hybrid of cases 0 and 2. As [9] points out, the means of calculating the total number of elements in a QHARS device for cases 1, 2, and 3, respectively, are as follows:

(6) $$D_{\text{T}}\left(M,\xi ,q_{M:ij}\right)=M\xi -\frac{\left(M+1\right)}{\xi }+\frac{1}{\xi }{\left(\xi q_{M:ij}^{\left(\text{approx}\right)}+1\right)}^{2^{-M}}+\frac{1}{\xi }\sum_{x=1}^{M}{\left(\xi q_{M:ij}^{\left(\text{approx}\right)}+1\right)}^{2^{-x}}$$

(7) $$D_{\text{T}}\left(M,\xi ,q_{M:ij}\right)=(2M-1)\xi +\frac{2}{\xi }{\left(\xi q_{M:ij}^{\left(\text{approx}\right)}+1\right)}^{2^{-M}}-\frac{2}{\xi }+2\sum_{x=2}^{M}\left[\frac{1}{\xi }{\left(\xi q_{M:ij}^{\left(\text{approx}\right)}+1\right)}^{2^{-x}}-\frac{1}{\xi }\right]$$

(8) $$D_{\text{T}}\left(M,\xi ,q_{M:ij}\right)=2\xi \sum_{x=1}^{M}\left[2^{\frac{x-H\left[(-1{)}^{x+1}\right]}{2}-1}\right]+\left(\frac{1}{\xi }{\left(\xi q_{M:ij}^{\left(\text{approx}\right)}+1\right)}^{2^{-M}}-\frac{1}{\xi }\right)*2^{\frac{M+H\left[(-1{)}^{M+1}\right]}{2}+H\left[(-1{)}^{M}\right]}+H\left[M-\frac{3}{2}\right]*\sum_{x=1}^{M-1}2^{\frac{x}{2}}*H\left[(-1{)}^{x}\right]*\left(\frac{1}{\xi }{\left(\xi q_{M:ij}^{\left(\text{approx}\right)}+1\right)}^{2^{-x}}-\frac{1}{\xi }\right).$$

These formulas will help extract the first variable, a metric of the divergence away from a neighborhood of desired resistances, but first, one must also define the second variable reflecting a pseudofractal complexity, since selecting certain design approaches has implications on recovering QHARS device utility in the event of a grounded branch failure. This second variable will be the MBD, which characterizes a fractal or pseudofractal via a ratio of its change in detail to change in scale [9]. This quantity is calculated via the box-counting method of vector representations of the topological drawings shown in figure 3(a), yielding the behavior in (b), whose linearity determines the value of the MBD. Example pseudofractals from the M=9 iterations of all four cases from figure 3(a) are plotted, and figure 3(c) shows a summary of the MBDs for each pseudofractal as a function of ξ, with two distinct curves for the same M=9 iterations and for the ideal case of the pseudofractal where the iterations approach infinity.

Now that the MBDs are determined, one may focus on the metric of divergence, namely, an exponential growth factor for the total elements in a QHARS device for each of the cases. This general way of correlating whether a design provides localized neighborhoods of resistances involves inspecting $D_{\text{T}}$ [9]. Rapid divergence or rate of change of $D_{\text{T}}$ as M→∞ implies that minor unintended modifications (such as the failure of a single grounded Hall element) are likely to result in large perturbations of the output QHARS device resistance.

Each case was examined by inspecting $D_{\text{T}}$ in figure 4(a) as a function of M and for a nominal value near 1GΩ. With each case, the set of curves for ξ={1,2,3,5,and10} are logarithmically normalized such that the minimum curve values are at 10 for easier comparison [9]. The true total number of devices are in the supplemental material [14]. In the first panel of figure 4(a), a dashed orange line is added for visual clarity and horizontally translated to determine the exponential coefficient, starting at the global minimum of the relevant curve (this is essentially the derivative of the adjusted $D_{\text{T}}$). These coefficients are determined by best fit to an exponential growth curve in OriginLab (see acknowledgments), and each fit parameter is associated with an error. Consistent with the results of [9]., cases 1 and 2 do not change as rapidly as cases 0 and 3, regardless of ξ. This observation can be explained by taking the limit of equations (6)–(8); that is, when M approaches infinity in cases 0 and 3, the growth is exponential, and for cases 1 and 2, the growth is linear.

With both variables now defined, one may calculate the Pearson correlation coefficient between the MBD of a pseudofractal (one of four cases) and the exponential coefficients in (a). Although the MBD and $D_{\text{T}}$ are calculated from theoretical frameworks, the resulting correlation is an empirical observation derived from the performance of these specific topological cases. This correlation is shown in figure 4(b) and yields 0.6398 ± 0.0077. Similar, but not identical to [9], these results suggest a trend wherein higher MBD topologies tend to correlate with a reduced probability of designing QHARS devices with highly localized output resistances. This observation is specific to the classes of pseudofractals investigated here. Also, one may state that pseudofractals exhibiting linear behavior are preferred if QHARS device designers wish to seek greater ease of access to a localized neighborhood of resistance values. Additional simulations for each of the four cases are provided in the supplemental material to further illustrate the divergence of a device output in the event of a grounded branch failure [14].

This work refines a recently developed mathematical framework for minimizing the number of elements in a QHARS device and addresses slight discrepancies between desired and actual resistance values that arise from approximations within the initial framework. The analysis is supported by data from an approximately 1GΩ QHARS device and prompts one to explore the benefits of other pseudofractal designs. By examining the MBD of these pseudofractals, one can correlate QHARS device complexity with device output resistance divergence from the desired neighborhood when subjected to grounded branch failure. This understanding provides significant advantages for designers of future QHARS device topologies.

## Supplementary Material

Supp1

Supplemental Material: Fabrication of Graphene-Based Quantum Hall Networks and Influences of Partial Star-Mesh Recursion available at https://doi.org/10.1088/2053-1591/ae7250/data1.

Supplementary material for this article is available online

## Figures and Tables

**Figure 1.**
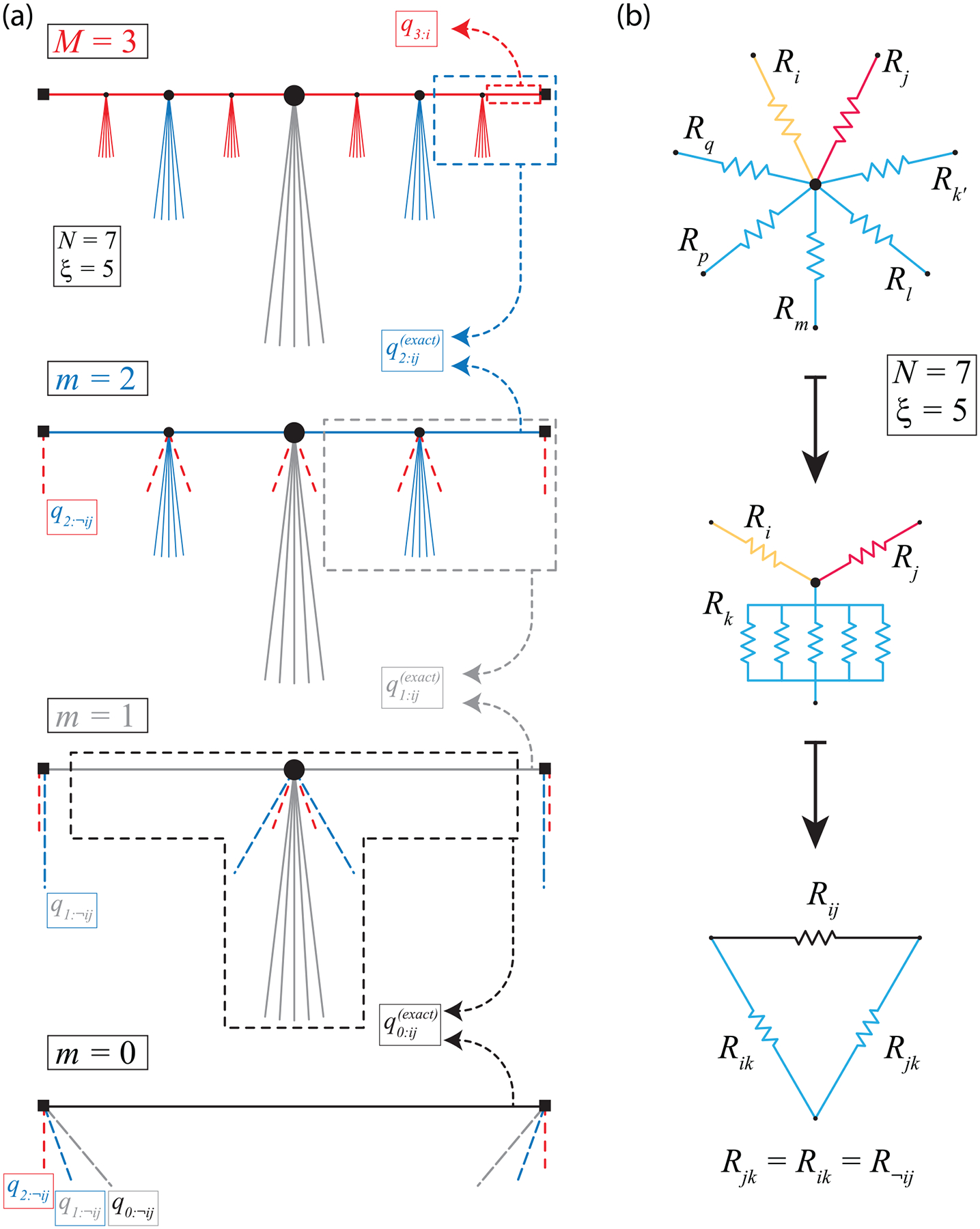
(a) This illustration serves to guide one’s intuition on how some virtual resistances $\left(q_{m:--ij}\right)$ contribute to the overall resistance network (with an example focused on a device design where M=3). After the full optimization process, the quantity $q_{M:i}$ always remains an exact number of elements, whereas every other form of q represents an *effective* resistance. With the inclusion of virtual resistances from every star-mesh transformation, calculating $q_{m<M:ij}^{\text{(exact)}}$ becomes more cluttered. (b) One approach to simplifying the manual procedure of recalculating $q_{0:ij}^{\text{(exact)}}$, which is the quantity one should expect to measure from the device, is to treat all ξ and branches as parallel branches in a single Y-Δ network.

**Figure 2.**
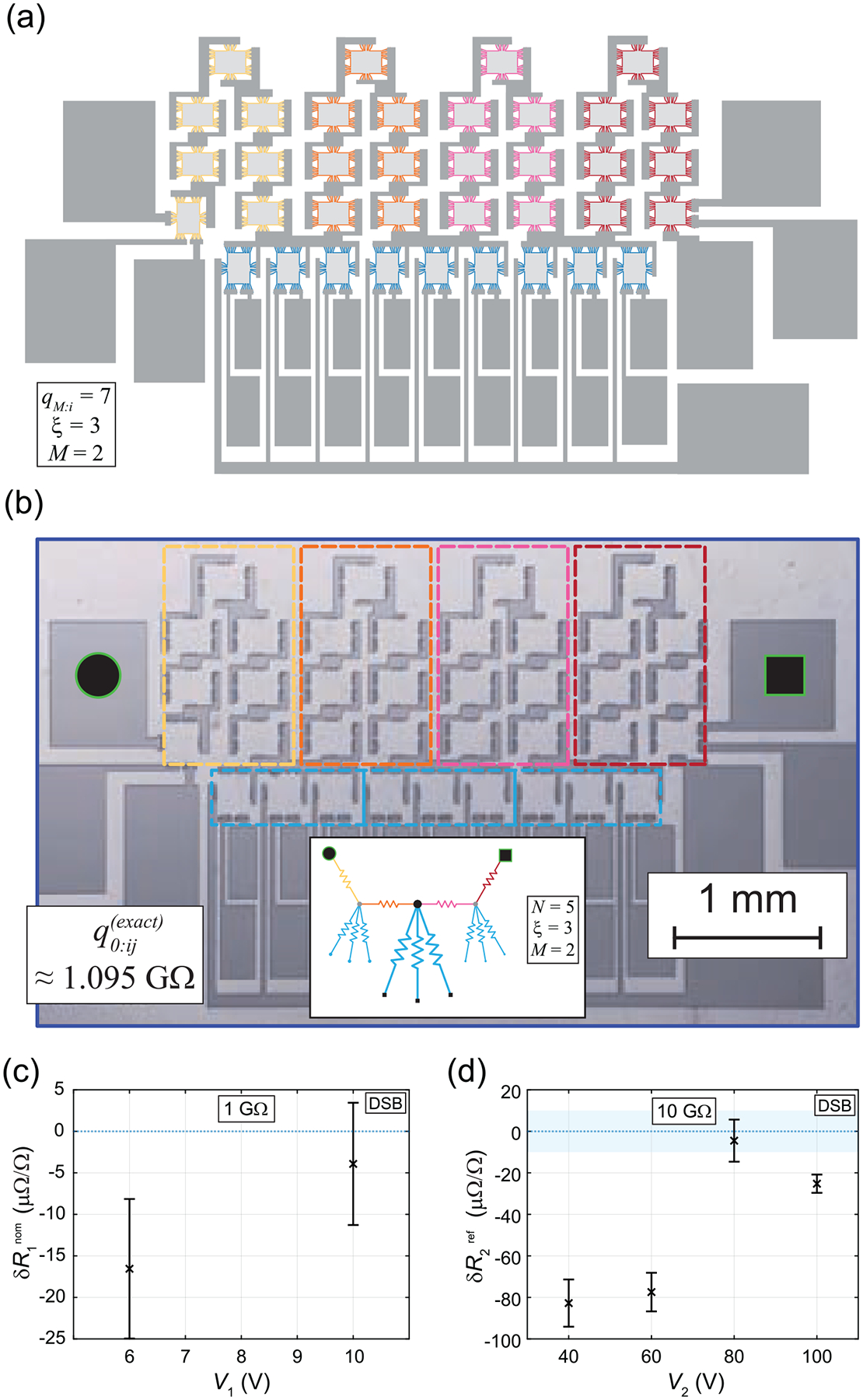
(a) QHARS device design layout with corresponding parameters M=2,ξ=3, and $q_{M:i}=7$. (b) Device post-fabrication optical image, overlaid by color-coded markers that match with those in the central inset showing a simplified network drawing presented in the style of [8]. (c) DSB data are shown to verify that the QHARS device is quantized (that is, each of its elements are successfully exhibiting the quantum Hall effect with high precision), with a standard resistor serving as the known quantity. The deviation from nominal $\delta R_{1}$ is centered around $q_{0:ij}^{\text{(exact)}}=1.095069\ldots \;\text{G}\Omega$. Error bars represent a 1σ uncertainty. (d) More DSB data denote the utility in using QHARS devices for calibrations at 10GΩ. The highlighted blue is the 1σ uncertainty on the value of the standard resistor and the data points are the determined value of the same resistor using the QHARS device, with error bars representing 1σ *uncertainty*.

**Figure 3.**
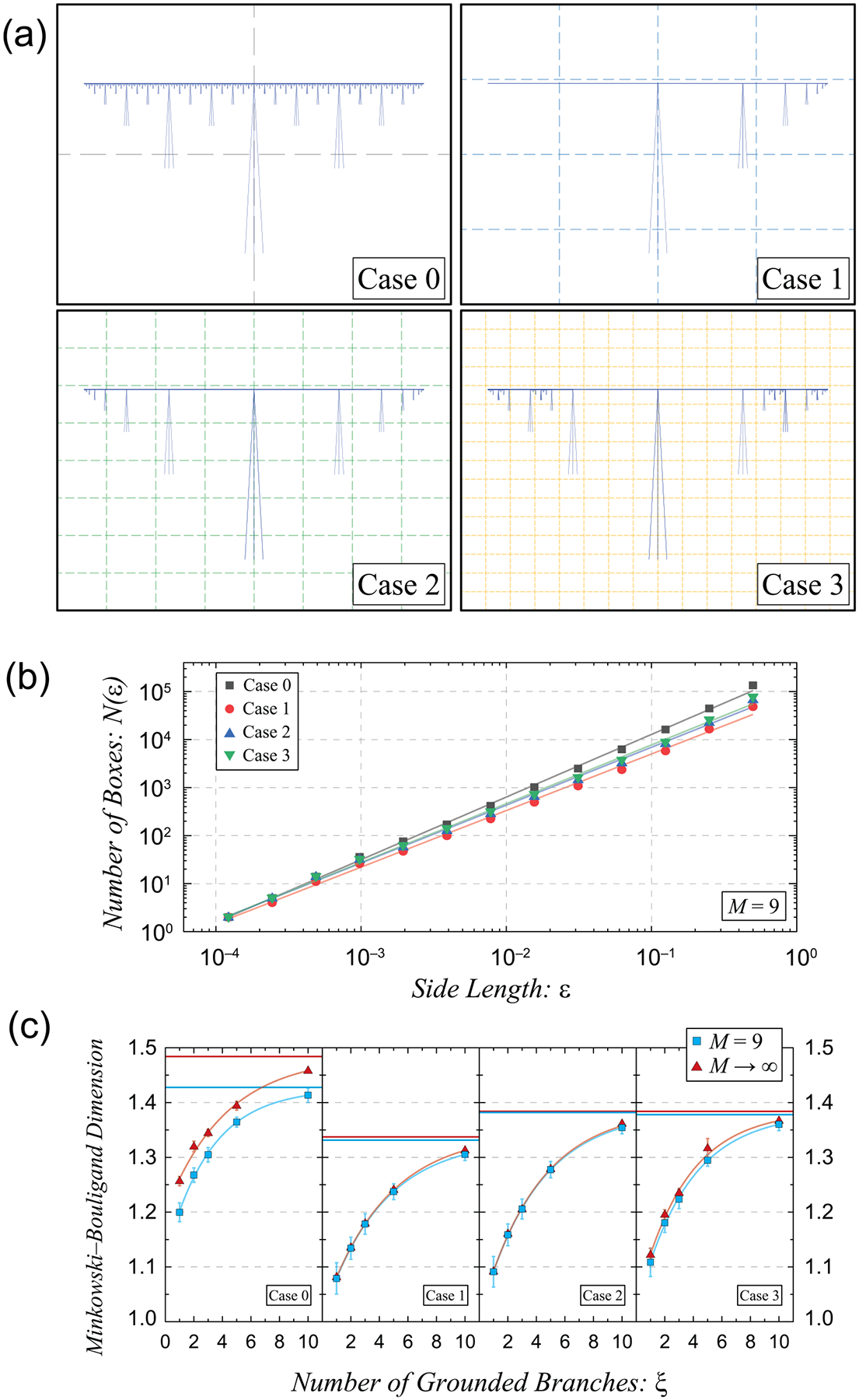
(a) This illustration depicts the four distinct pseudofractals used for the cases in the relevant calculations. Each panel is accompanied by an example overlay of a single box-counting computation, where the boxes containing the pseudofractal are tracked as a function of box size. (b) The prior computations are tabulated and plotted on a log–log plot, whose resulting curves may be fit to a line whose slope reveals the Minkowski–Bouligand dimension of the relevant pseudofractal. Example pseudofractals from the M=9 iterations of all four cases from (a) are plotted. (c) A summary of the MBDs for each pseudofractal are shown as a function of ξ, with two distinct curves for the same M=9 iterations (in blue) and for the ideal case of the pseudofractal where the iterations approach infinity (in red).

**Figure 4.**
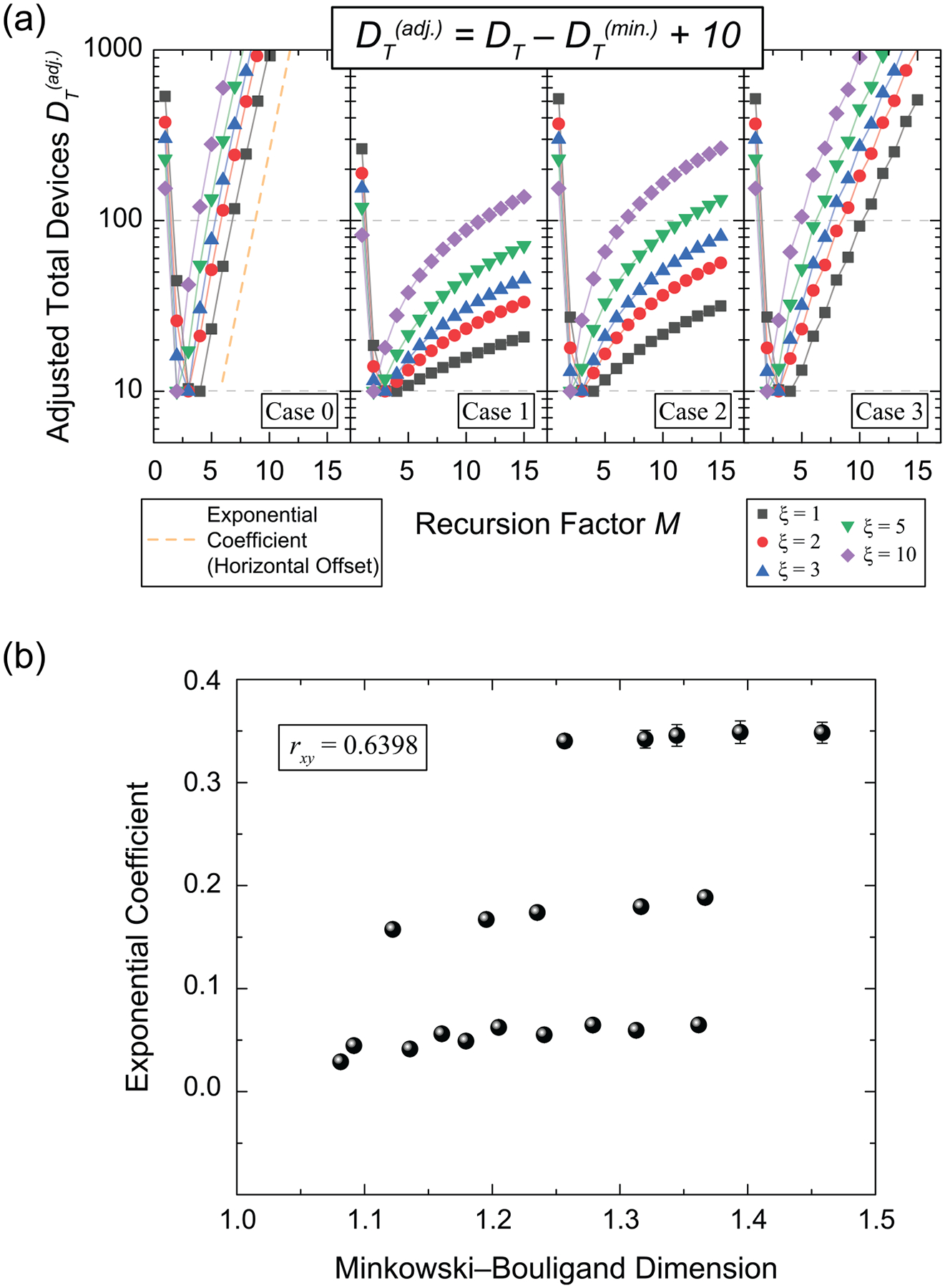
(a) Calculations for the adjusted total devices as a function of recursion factor (M) are performed for each of the four cases of pseudofractal design. The target resistance was 1GΩ, and all plots are logarithmically normalized such that the minimum values are at 10 for easier comparison. The true total number of devices are in the supplemental material [14]. A horizontally offset exponential growth fit is imposed in the first panel. (b) The Pearson correlation coefficient is determined between the calculated Minkowski–Bouligand Dimension and the exponential coefficients in (a). The error bars indicate 1σ uncertainty, which were determined explicitly from the standard deviation of the slope as providing by the fitting software in OriginLab (see acknowledgements for commercial disclaimer).

## Data Availability

Data that support the findings of this study are available from the corresponding author upon reasonable request. All data that support the findings of this study are included within the article (and any supplementary files).
