# Supplementary material for: Fabrication of graphene-based quantum Hall networks and influences of partial star-mesh recursion
Source: Mater Res Express. Author manuscript; Available in PMC 2026 Jun 9. (PMC13244287; doi:10.1088/2053-1591/ae7250)
Supplement: Supp1 [file NIHMS2181441-supplement-Supp1.pdf]

# Supplemental Material: Fabrication of Graphene-Based Quantum Hall Networks and Influences of Partial Star-Mesh Recursion

D. S. Scaletta,[1] N. T. M. Tran,[2,3] M. Musso,[4] V. Ortiz Jimenez,[2] H. M. Hill,[2] D. G. Jarrett,[2] M. Ortolano,[4] C. A. Richter,[2] D. B. Newell,[2] and A. F. Rigosi[2,a)]

[1]*Department of Physics, Mount San Jacinto College, Menifee, California 92584, USA*

[2]*Physical Measurement Laboratory, National Institute of Standards and Technology (NIST), Gaithersburg, Maryland 20899, USA*

[3]*Joint Quantum Institute, University of Maryland, College Park, Maryland 20742, USA*

[4]*Department of Electronics and Telecommunications, Politecnico di Torino, Torino 10129, Italy*

[a)] Author to whom correspondence should be addressed. email: afr1@nist.gov

Table of Contents:

1. Additional context for Dual Source Bridges and calibrations
2. Logarithmic Normalization Values for Figure 4
3. Simulations of Grounded Branch Failures

**1. Additional context for Dual Source Bridges and calibrations**

For context, the DSB has been used as the primary method for the measurement of high resistance ranges. The bridge is an adapted Wheatstone bridge, which is based on the automated high resistance measurement approach initially proposed by Henderson (see main text Ref. [13]). The DSB is configured with two voltage sources in the main ratio arms, substituting two corresponding resistors in the Wheatstone bridge and forming a voltage ratio bridge. When the bridge is balanced, the arm containing the unknown resistance value $R_x$ can be determined via $R_x = R_s \frac{V_x}{V_s}$, where $R_s$ is a standard resistor and $V_x$ and $V_s$ are the applied voltages across $R_x$ and $R_s$, respectively.

Each standard resistor was calibrated by a conventional traceability chain involving the use of a graphene-based quantized Hall resistance standard that is approximately 12.9 kΩ, a cryogenic current comparator, and guarded Hamon transfer standards to reach the two high resistance values (see main text Ref. [12]).

**2. Logarithmic Normalization Values for Figure 4**

Table 1-SM. To better compare the behavior of the total device count in the main text, each curve was logarithmically normalized to its global minimum. This table summarizes those values of $D_T^{(min)}$, rounded to the nearest integer:

| 1 GΩ | $D_T^{(min)}$ | | | |
|---|---|---|---|---|
| Case | 0 | 1 | 2 | 3 |
| ξ = 1 | 31 | 302 | 49 | 49 |
| ξ = 2 | 28 | 215 | 36 | 36 |
| ξ = 3 | 30 | 178 | 34 | 34 |
| ξ = 5 | 34 | 144 | 34 | 34 |
| ξ = 10 | 41 | 114 | 41 | 41 |

### 3. Simulations of Grounded Branch Failures

To test, in practice, how each pseudofractal correlates with a divergent behavior of the QHARS output, several examples of grounded branch failures were simulated with LTspice.

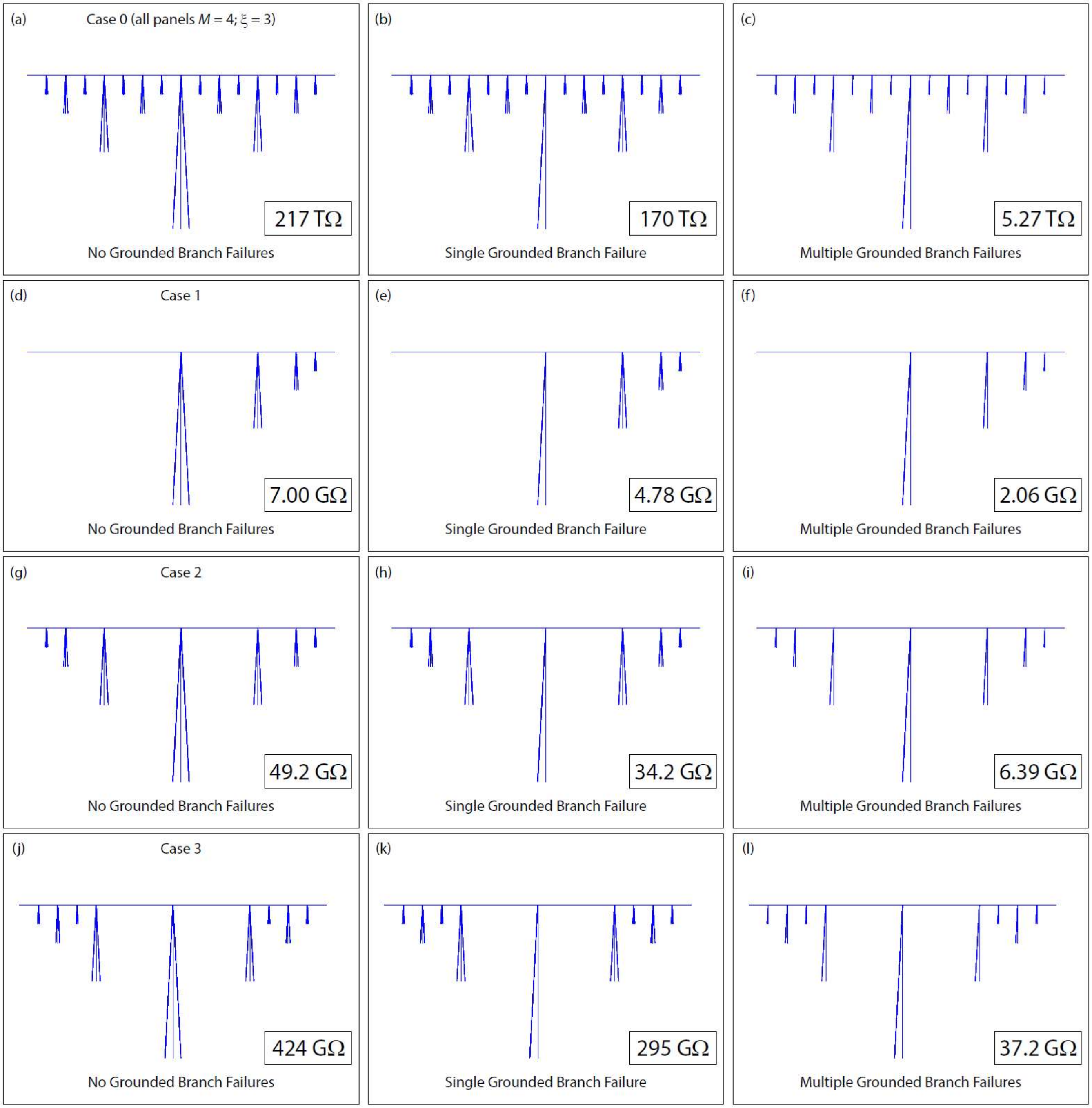


FIG 1-SM. Example simulations in LTspice to obtain grounded branch failure impacts on a QHARS device.
